# Pembrolizumab-Induced Adrenal Crisis Indicated by Increased 18F-Fluoro-D-Deoxyglucose Uptake in Bilateral Adrenal Glands Prior to Onset

**DOI:** 10.7759/cureus.108272

**Published:** 2026-05-04

**Authors:** Chikara Kase, Kenta Takahashi, Jun Ogino, Sho Watanabe, Akira Toriihara

**Affiliations:** 1 Radiology, Asahi General Hospital, Chiba, JPN; 2 Obstetrics and Gynecology, Asahi General Hospital, Chiba, JPN; 3 Diabetes and Metabolic Diseases, Asahi General Hospital, Chiba, JPN; 4 PET Imaging Center, Asahi General Hospital, Chiba, JPN

**Keywords:** adrenal crisis, adrenal insufficiency, fdg-pet/ct, immune-related adverse events, pembrolizumab

## Abstract

We present a case of adrenal crisis induced by pembrolizumab in a 51-year-old female patient diagnosed with cervical cancer (cT2bN1M1, cStageIVB). Immune checkpoint inhibitors are known to trigger serious immune-related adverse events (irAEs), such as primary adrenal insufficiency. The patient exhibited nonspecific symptoms such as loss of appetite, fever, and shock, indicating pituitary and adrenal insufficiency attributed to pembrolizumab. ^18^F-fluoro-D-deoxyglucose (FDG) positron emission tomography/computed tomography (PET/CT), performed four days prior to admission, had revealed increased diffuse FDG uptake in bilateral adrenal glands, suggesting potential adrenal crisis. This case underscores that those PET/CT findings may represent an initial indicator of following adrenal irAEs, facilitating early diagnosis and intervention.

## Introduction

Immune checkpoint inhibitors (ICIs) are widely used in patients with various malignant tumors, such as cervical cancer. ICIs enhance antitumor activity by inhibiting the binding of immune checkpoint receptors, such as programmed cell death 1 (PD-1), to their ligands [1]. Although ICIs are effective in triggering tumor immune responses to combat cancer, they may also lead to immune-related adverse events (irAEs) through autoimmune pathways [1]. ^18^F-fluoro-D-deoxyglucose (FDG) positron emission tomography/computed tomography (PET/CT) is a useful test for evaluating the therapeutic effect of immunotherapy, and it is also being studied as a test that can detect adverse events caused by immunotherapy [2,3].

irAEs can manifest as generalized immune activation issues, such as diarrhea and rash, or as organ-specific concerns such as colitis and pneumonia [1]. Among the latter are endocrine-related adverse events. While hypopituitarism and hypothyroidism are major types of endocrine irAEs [4,5], adrenal insufficiency is noted as one of the rarest yet most critical adverse events, potentially leading to serious outcomes.

In this report, we present the clinical course and radiological findings of a case of primary adrenal insufficiency occurring during pembrolizumab therapy for cervical cancer.

## Case presentation

A 51-year-old female patient visited the emergency department, complaining of a month-long loss of appetite and diarrhea, accompanied by a fever that started the previous day. She had been diagnosed with cervical cancer with supraclavicular lymph node metastasis and multiple lung metastases (cT2bN1M1, cStageIVB) nine months before. She had received three cycles of chemotherapy involving paclitaxel, carboplatin, and bevacizumab, achieving a partial response. Subsequently, pembrolizumab, an anti-PD-1 antibody, at a dose of 200 mg every three weeks, was incorporated into her treatment five months earlier, resulting in a total of 10 courses of immunochemotherapy.

During the emergency room evaluation, the patient's physical examination indicated hypotension with a blood pressure of 87/67 mmHg, tachycardia with a pulse rate of 130 beats per minute, and tachypnea with a respiratory rate of 26 breaths per minute. The percutaneous oxygen saturation was at 98% in room air. The bowel sounds were hyperactive. Laboratory tests showed hyponatremia, hypokalemia, and hypochloremia, alongside elevated levels of C-reactive protein (CRP). White blood cell count was 7,400/µL, and the eosinophil percentage was 0.4%. Urinalysis revealed ketonuria (Table 1).

**Table 1:** Laboratory data

Category	Parameter	Patient Value	Reference Range
Blood Test	Sodium	117 mEq/L	138-145 mEq/L
	Potassium	3.1 mEq/L	3.6-4.8 mEq/L
	Chloride	84 mEq/L	101-108 mEq/L
	C-reactive protein	15.61 mg/dL	<0.14 mg/dL
	Glucose	131 mg/dL	73-109 mg/dL
Urinalysis	Ketones	(4+)	-

Contrast-enhanced CT demonstrated small bowel dilatation and fluid accumulation without any signs of obstruction (Figure 1).

**Figure 1:**
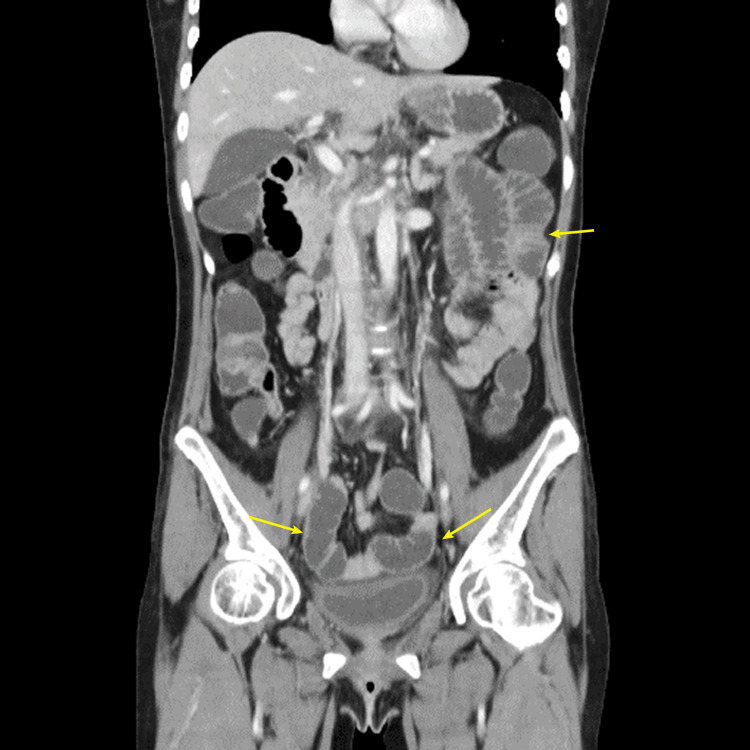
Coronal contrast-enhanced CT at admission showing small bowel dilatation and fluid accumulation (arrows)

With fluid resuscitation, both the blood pressure and pulse rate improved, leading to the patient’s admission for further fluid therapy and correction of electrolytes. Colonoscopy after admission revealed a reduction in the length of the intestinal villi (Figure 2).

**Figure 2:**
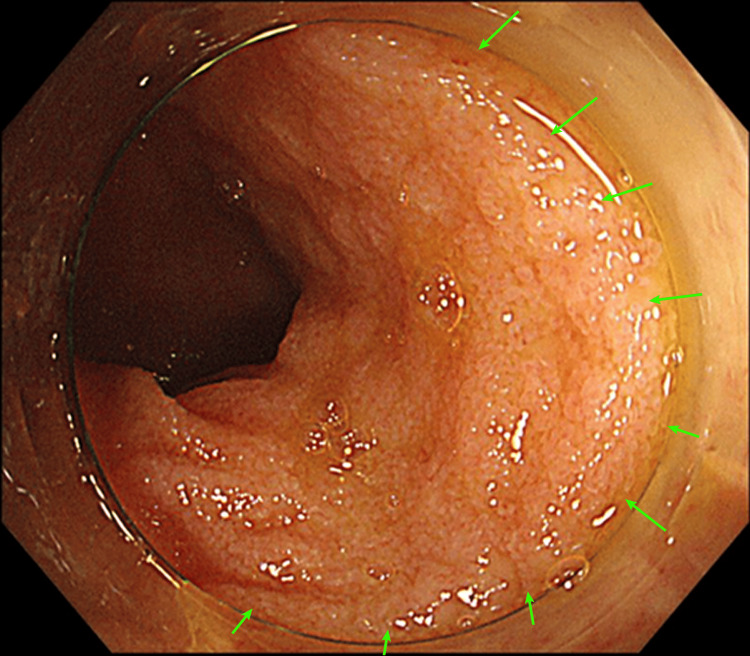
Colonoscopy showing shortening of length of intestinal villi (arrows)

To more accurately elucidate the pathophysiology, a random biopsy was performed. The obtained specimen showed apoptosis of the intestinal epithelium, compatible with the diagnosis of immune-related enteritis induced by pembrolizumab. Concerning electrolyte imbalances, both plasma adrenocorticotropic hormone (ACTH) and serum cortisol levels were found to be low at the time of admission. Such a trend had been noted in an examination conducted one month earlier (Table 2).

**Table 2:** Hormone level trends ACTH: adrenocorticotropic hormone

Parameter	Two months before admission	One month before admission	At admission	Reference Range
Cortisol (µg/dL)	16.4	0.4	3.0	6.24-18
ACTH (pg/mL)	73	2.7	<1.5	7.2-63.3
White blood cell (/µL)	5,800	8,400	5,200	3,300-8,600
Neutrophil	51.7%	45.4%	62.4%	38.3-74.7%
Lymphocyte	37.4%	34.8%	24.2%	21.2-51%
Monocyte	6.1%	8.1%	10.1%	2.7-8.0%
Eosinophil	0.3%	0.8%	0.8%	0.2-2.0%
Basophil	4.5%	10.9%	2.5%	0.2-8.4%
Hemoglobin (g/dL)	13.1	13.6	11.7	11.6-14.8
Platelet (/µL)	230,000	288,000	136,000	158,000-348,000
Total protein (g/dL)	6.9	6.7	4.8	6.6-8.1
Albumin (g/dL)	4.1	3.8	2.5	4.1-5.1
Blood urea nitrogen (mg/dL)	10	5	6	8-20
Creatinine (mg/dL)	0.61	0.57	0.5	0.46-0.79
Sodium (mEq/L)	132	121	124	138-145
Potassium (mEq/L)	4.1	3.8	3.8	3.6-4.8
Chloride (mEq/L)	99	83	95	101-108
C-reactive protein (mg/dL)	0.03	1.27	19.82	<0.14

Subsequently, endocrine tests were performed (Table 3).

**Table 3:** Endocrine test findings CRH: corticotropin releasing hormone; LHRH: luteinizing hormone-releasing hormone; TRH: thyrotropin releasing hormone; LH: luteinizing hormone; FSH: follicle stimulating hormone; TSH: thyroid stimulating hormone; GH: growth hormone

Test	Parameter	0 min.	15 min.	30 min.	45 min.	60 min.	90 min.	120 min.	Reference Range
Rapid ACTH stimulation test	ACTH (pg/mL)	1.8	-	-	-	-	-	-	6.24-18
	Cortisol (µg/dL)	0.4	-	3.2	-	4.4	-	-	7.2-63.3
Combined anterior pituitary stimulation test (CRH, LHRH, TRH)	ACTH (pg/mL)	2.1	2.3	1.7	-	2.0	1.7	2.2	6.24-18
	Cortisol (µg/dL)	0.6	0.6	0.5	-	0.5	0.5	0.4	7.2-63.3
	LH (mIU/mL)	30.10	-	70.00	-	90.80	85.60	84.50	-
	FSH (mIU/mL)	55.10	-	64.30	-	77.70	78.80	81.10	-
	PRL (ng/mL)	25.90	-	213.00	-	134.00	87.80	57.60	-
	TSH (µIU/mL)	2.31	-	12.20	-	9.92	6.99	5.16	0.50-5.00
	Free T_3_ (pg/mL)	3.49	-	-	-	-	-	-	2.30-4.30
	Free T_4_ (ng/dL)	1.61	-	-	-	-	-	-	0.90-1.70
Insulin tolerance test (glucose administered at 28min.)	Glucose (mg/dL)	85	67	51	113	85	69	76	73-109
	ACTH (pg/mL)	<1.5	<1.5	<1.5	<1.5	<1.5	<1.5	<1.5	6.24-18
	Cortisol (µg/dL)	1.83	1.74	1.77	1.7	1.65	1.66	1.67	7.2-63.3
	GH (ng/mL)	1.69	0.92	2.79	8.43	6.09	4.50	6.04	0.13-9.88

A rapid ACTH stimulation test resulted in poor elevation of cortisol. Furthermore, a combined anterior pituitary stimulation test and an insulin tolerance test revealed no elevation of ACTH or cortisol, which suggested potential pituitary and adrenal insufficiencies. These findings were attributed to endocrine adverse events associated with pembrolizumab.

Four days before admission, the patient had undergone FDG-PET/CT to assess the activity of cervical cancer lesions. During this examination, increased FDG uptake was noted in bilateral adrenal glands without any enlargement on CT (Figure 3).

**Figure 3:**
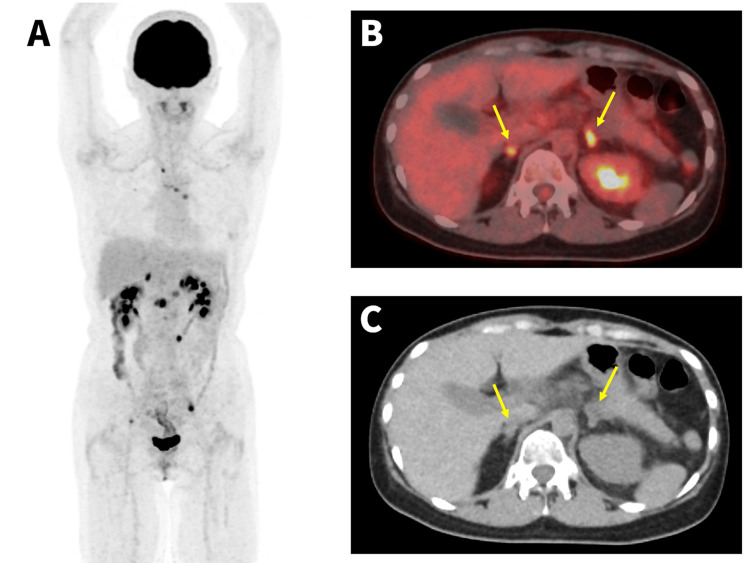
FDG-PET/CT showing increased FDG uptake in bilateral adrenal glands (A-B). However, CT image did not reveal any enlargement (C). (A) Maximum intensity projection (MIP) image in which multiple lymph node metastases and lung metastases were detected as nodular FDG uptakes; (B) axial fusion image showing bilateral adrenal glands which showed diffuse elevated FDG uptake (arrows); (C) axial CT image showing bilateral adrenal glands which showed diffuse elevated FDG uptake (arrows).

Given the patient’s symptoms of anorexia and others at that time, those findings were considered to indicate adrenal insufficiency. Pituitary magnetic resonance imaging (MRI) was performed during the hospitalization, which revealed no abnormalities suggestive of pituitary inflammation.

Upon admission, the patient experienced another episode of shock, which was managed successfully with adrenergic medications and hydrocortisone (200 mg per day). Subsequently, the hydrocortisone dosage was gradually reduced, and she was discharged nine days post admission. The patient died 18 months later due to the progression of cervical cancer.

## Discussion

Endocrine-related adverse events, a subset of irAEs, encompass conditions such as pituitary inflammation, thyroid dysfunction, type 1 diabetes, and adrenal insufficiency [4,5]. Pituitary inflammation is more commonly associated with anti-cytotoxic T-lymphocyte-associated antigen 4 (CTLA-4) therapies, while thyroid dysfunction is linked to anti-PD-1 therapies. Primary adrenal insufficiency is particularly rare, with a meta-analysis estimating an incidence rate of 0.8% when using pembrolizumab alone [5].

The clinical manifestation of ICI-induced primary adrenal insufficiency closely resembles that of Addison's disease, with acute adrenal insufficiency often resulting in severe outcomes. Symptoms such as anorexia, nausea, vomiting, abdominal pain, and weakness are nonspecific, frequently causing delays in diagnosis. Hyponatremia, hyperkalemia, and hypercalcemia are occasionally observed. Definitive diagnosis is based on decreased morning cortisol levels. Concurrently, elevated and decreased ACTH levels suggest primary and secondary adrenal insufficiency, respectively [5]. In the present case, the combination of enteritis as irAEs and adrenal insufficiency resulted in shock and adrenal crisis. Measurements of random serum cortisol and plasma ACTH during hospitalization confirmed the diagnosis of adrenal insufficiency, facilitating the timely initiation of glucocorticoid therapy. Based on endocrine test results and FDG/PET-CT findings, it is possible that primary and secondary adrenal insufficiency coexist. There are reports of cases where pituitary inflammation and adrenalitis coexisting as irAEs were demonstrated pathologically [6]; a similar pathology may be considered in this case.

Radiological findings of primary adrenal insufficiency due to irAEs include bilateral adrenal enlargement and increased FDG uptake suggestive of inflammation [7-10]. Such findings can also be observed in pheochromocytoma and adrenal hyperplasia associated with Cushing's syndrome [11]. In this case, vasovagal reflex, a common cause of increased FDG uptake in bilateral adrenal glands [12], was suspected during image analysis. Nonetheless, the patient had not complained of any discomfort throughout the FDG injection process. Furthermore, no change in size or shape was observed in adrenal glands throughout the course of cervical cancer (Figure 4), leading us to consider adrenal metastases unlikely [9].

**Figure 4:**
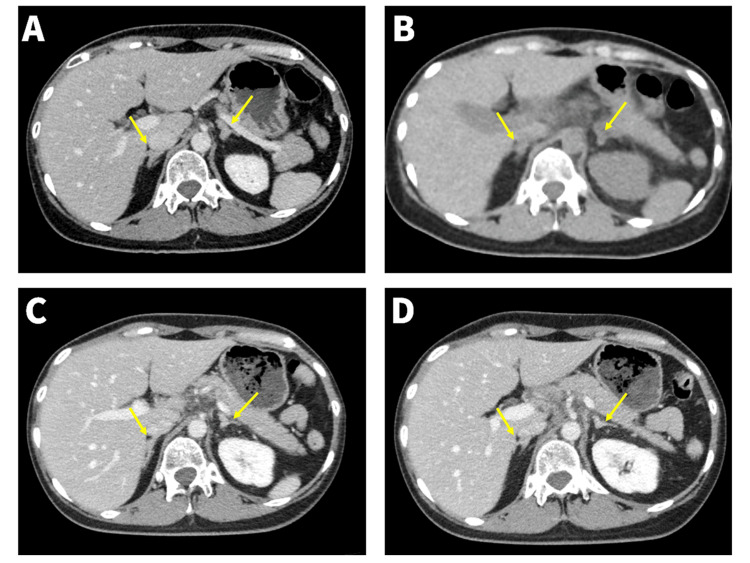
Axial CT findings during the course of cervical cancer. (A) Six months before the FDG-PET; (B) CT performed concurrently with FDG-PET; (C) One month after the FDG-PET; (D) Four months after the FDG-PET. All arrows depict both adrenal glands.

Even though repeated PET/CT was not performed and it was not possible to compare adrenal FDG uptake over time, those findings may aid in the early detection of adrenal insufficiency, a rare form of irAEs.

## Conclusions

We described the clinical course of a patient experiencing adrenal crisis triggered by ICI, where FDG-PET/CT may have reflected adrenal inflammation preceding adrenal crisis. While the precise mechanisms underlying ICI-induced endocrine adverse events remain unclear, it is anticipated that the number of affected patients will increase as ICIs are more widely used. When increased FDG uptake in both adrenal glands is detected on FDG-PET/CT in patients receiving immunochemotherapy, the possibility of adrenal insufficiency due to irAEs should be taken into account for rapid and appropriate patient management, although asymptomatic, in the course of PET/CT.
